# Enteropathogenic *Escherichia coli*, *Samonella*, *Shigella *and *Yersinia*: cellular aspects of host-bacteria interactions in enteric diseases

**DOI:** 10.1186/1757-4749-2-8

**Published:** 2010-07-22

**Authors:** Roberta Souza dos Reis, Fabiana Horn

**Affiliations:** 1 Departamento de Biofísica, Universidade Federal do Rio Grande do Sul, P.O. Box 15005, 91501-970, Porto Alegre, Brazil; 2 Departamento de Microbiologia, Universidade Federal do Rio Grande do Sul, Av. Sarmento Leite, 500, 90050-170, Porto Alegre, Brazil

## Abstract

A successful infection of the human intestine by enteropathogenic bacteria depends on the ability of bacteria to attach and colonize the intestinal epithelium and, in some cases, to invade the host cell, survive intracellularly and disseminate from cell to cell. To accomplish these processes bacteria have evolved an arsenal of molecules that are mostly secreted by dedicated type III secretion systems, and that interact with the host, subverting normal cellular functions. Here we overview the most important molecular strategies developed by enteropathogenic *Escherichia coli, Salmonella enterica*, *Shigella flexneri*, and *Yersinia enterocolitica *to cause enteric infections. Despite having evolved different effectors, these four microorganisms share common host cellular targets.

## Introduction

Diarrhea-causing pathogens employ a variety of sophisticated strategies to colonize the intestinal epithelium. In essence, ingested pathogens have evolved the abilities to: (1) resist non-specific host defenses, such as stomach acidity, peristalsis, mucosal cell exfoliation, intestinal mucins, and bacteriocins [1]; (2) adhere to intestinal epithelia; and (3) ultimately colonize the epithelia. Colonization may, or may not, involve cellular invasion. When cellular invasion occurs, it can be followed either by intracellular multiplication and spread of the bacteria to other tissues, or by bacterial persistence.

Although bacterial pathogens employ different strategies, they share common targets in the host cell and often cause the same cellular responses. Virulence factors act either from the extracellular milieu mimicking cell ligands, or are injected into the intracellular milieu, where they act in concert to manipulate signaling pathways, for example to subvert the plasticity of the cytoskeleton, interrupt the endocytic traffic, and, in some cases, mediate resistance to phagocytosis. The host cell, in turn, defends itself against infection by initiating an inflammatory response and by altering the intestinal fluid balance in order to extrude the unwanted bacteria, thus causing diarrhea. Hence, the success of infection depends on host cell-bacteria interactions, and in each step bacteria exploit the target cell machinery for their own benefit.

## Adhesion

Adhesion is the preliminary step in the infectious process and requires strong and specific interactions. Table 1 lists the best characterized adhesins in the diarrhea-causing bacteria discussed in this review, and their cognate host-cell receptors. Bacterial attraction to intestinal epithelia is also favored by local environmental factors such as alkaline milieu, humidity and anaerobiosis.

**Table 1:** Bacterial adhesive structures and their cognate receptors

Pathogen	Adhesin	Receptor	Effect on host cell	Reference
EPEC	BFP EspA Intimin	Not fully elucidated Not fully elucidated Tir	Activation of T3SS^1 ^and formation of A/E^2 ^lesions and changes of epithelial cell morphology	[2,4]

*Salmonella* sp.	FimA FimH	Mannosylated proteins in epithelial cells	Activation of T3SS and transport of effector proteins necessary for invasion	[14]

*Shigella *sp.	Complex IpaB/C IpaB	Integrin α_5 _β_1_ CD44 (natural receptor of hyaluronic acid)	Activation of T3SS and transport of effector proteins necessary for invasion	[18,19]

*Yersinia* sp.	Invasin YadA	β_1 _integrins (natural receptor of fibronectin)	Activation of T3SS and transport of effector proteins necessary for invasion and/or apoptosis	[44]

1. T3SS: Type III Secretion System.

2. A/E lesion: **a**ttaching/**e**ffacing lesion.

## Enteropathogenic *Escherichia coli *(EPEC)

**Figure 1:**
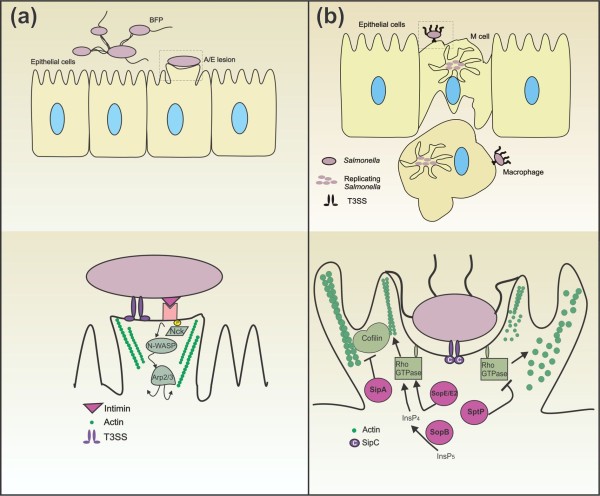
**Pathogenic mechanisms of (a) enteropathogenic *E*. *coli *(EPEC) and (b) *Salmonella enterica***. **(a) **EPEC contact gut epithelial cells, produce BFP (upper panel), and activate their T3SS (lower panel). The bacterium translocates the receptor for its adhesin, intimin, called Tir, through the T3SS into the host cell cytoplasm; the interaction between intimin and Tir promotes tight adhesion. After being phosphorylated by host kinases, the Tir binds Nck, which activates N-WASP, which in turn activates Arp2/3, leading to actin nucleation and formation of a pedestal beneath the bacterium. **(b) ***Salmonella *interacts with M cells (upper panel), activates its T3SS and translocates SipA and SipC. SipC localizes to the plasma membrane, where it aids in the translocation of other *Salmonella *effectors and initiates actin nucleation. The C-terminal domain nucleates actin and the N-terminal domain of SipC bundles it, anchoring the resulting actin filaments to the cell surface below the bacterium. The injected SipA acts in synergy with SipC, as SipA binds to and stabilizes the F-actin filaments, and blocks the action of ADF/cofilin (lower panel). SopE1, SopE2 and SopB activate the RhoGTPases that regulate actin polymerization; SopE1 and SopE2 do so directly by acting as GEFs, and SopB indirectly by interfering with inositol phosphate metabolism. The activated RhoGTPases induce cytoskeletal rearrangements that result in bacterial uptake. SptP then switches off the RhoGTPases, and the eukaryotic cell regains its normal shape. *Salmonella *replicates inside the vacuole (upper panel).

In response to the first contact with an epithelial cell, typical EPEC produce adhesive structures called bundle-forming pili (BFP) and form microcolonies that increase bacterial resistance to host defenses and are involved in the localized adherence pattern [2] (Fig. 1a, upper panel). Colonization of the intestinal epithelia by EPEC also involves activation of the T3SS. Type III Secretion Systems are essential Gram-negative virulence determinants believed to mediate the transport of virulence factors from the bacterial cytoplasm into host cells. The four pathogens discussed in this review secrete their virulence factors through a T3SS. Each complex is composed of more than twenty proteins, including integral membrane proteins, chaperones and accessory proteins. They are divided into two substructures: a bacterial membrane-embedded base and an extracellular needle-like filament, often called a translocon, through which effector proteins are injected [3]. Transport of effector proteins from the bacterial cytoplasm to the periplasmic space is accomplished at the expense of ATP. Once in the host cell cytoplasm, these effector proteins interact with eukaryotic pathways to alter host cell signal transduction.

Most T3SS-secreted effector proteins are encoded by a pathogenicity island called ***l**ocus *of **e**nterocyte **e**ffacement (LEE). EPEC T3SS translocon is constituted by **E**PEC **s**ecreted **p**rotein **A **(EspA), one of the first bacterial proteins secreted by the bacteria, that forms a filamentous extension from the bacterium to the surface of microvilli and acts in conjunction with EspB and EspD to form a pore structure in the host cell. The translocon permits translocation of other LEE effector proteins. The intimate adherence of EPEC - there is only 10 nm between bacterium and enterocyte - is promoted by an adhesin called intimin [2]. Intimin is an outer-membrane protein encoded by the *eae *gene in the LEE, and shares functional and structural homologies to the *Yersinia pseudotuberculosis *invasin protein [4,5]. The intimin receptor (Tir), also encoded by the LEE, is translocated by the T3SS and inserted into the host cell membrane, a dimer of Tir binds two intimin molecules [4]. Apart from its receptor function, Tir is phosphorylated on tyrosine residues by redundant host kinases [6], and this phosphorylation depends on Esps and T3SS [5]. Once phosphorylated, Tir binds Nck, a host protein that recruits and activates the amino-terminal domain of the Wiskott-Aldrich syndrome protein (N-WASP), which in turn recruits and activates actin-related protein 2/3 (Arp2/3). This results in actin filament nucleation^1 ^(see Appendix), leading to assembly of pedestal-like structures beneath the bacterium, typical of EPEC and enterohemorragic *E. coli *(EHEC), and formation of an **a**ttachment/**e**ffacement (A/E) lesion [7] (Fig. 1a, lower panel). Other effectors involved in microvilli effacement and pedestal formation are Map, EspF, EspG, EspH and EspZ, highlighting the complexity of these pathogenic processes [8]. Although the phosphorylation on tyrosine residues is sufficient for actin nucleation and lesion formation, Tir is also phophorylated on serine residues by host protein kinase A and this seems to be necessary for efficient pedestal formation [9]. Cholesterol in the host cell membrane also has a crucial function in EPEC adhesion, since cholesterol depletion reduces bacterial adherence and A/E lesion formation [10].

The diarrhea observed in EPEC infections probably results from several mechanisms, including loss of absorptive surface due to microvilli effacement, increased intestinal permeability and active ion secretion. Nevertheless, it is not known if these mechanisms result from the action of EPEC virulence effectors or are consequences of host-cell responses to bacterial adherence [7]. A report indicates that EPEC is able, in a T3SS-dependent mechanism, to quickly inhibit *in vitro *sodium/glucose cotransporter (SGLT-1) activity even before microvilli effacement and pedestal formation. This is a key diarrheagenic mechanism, given that SGLT-1 is responsible for the daily uptake of approximately 6 liters of fluids from the intestine [8]. Another report, which employed the A/E pathogen *Citrobacter rodentium *in a mouse infection model, proposed that the localization of aquaporins (water channels) in colonocytes of the infected mice is altered during infection, in a process partially dependent on EspF and EspG [11]. For both EPEC and *C. rodentium*, there is growing evidence that these pathogens target and disrupt epithelial tight junctions, thus contributing to the loss of ions and water during diarrhea [12]. Moreover, using the *C. rodentium *mouse model, Higgins and colleagues (1999) [13] have shown that intimin plays a dual role in EPEC pathogenesis: in addition to its function as a cellular ligand, it drives a Th1 pro-inflammatory response resulting in mucosal thickening and crypt hyperplasia. Throughout the course of the infection, EPEC remain extracellular and the hyperplasia seems to offer sufficient area for new colonization.

### Salmonella enterica

The initial interaction between *Salmonella *and the gut epithelium is believed to occur preferentially through M (**M**icrofold) cells, either from Peyer patches or from solitary intestinal lymphoid tissue (SILT), which is scattered along the small intestine. M cells are follicle-associated epithelium (FAE) cells that function in antigen sampling; their basolateral surface is invaginated to form a pocket-like structure, to which macrophages and lymphocytes can migrate and where they interact with antigenic particles.

There is evidence that *Salmonella *sp. fimbriae play an important role in attachment to the gut mucosa. Type 1 fimbriae are the most common adhesins in gram-negative bacteria; FimA and FimH are encoded by the *fim *operon, and the *Salmonella *genus contains at least another ten operons that encode fimbrins which, when expressed, act like adhesins [14]. Mutants in which genes involved in fimbrin biosynthesis were inactivated had their virulence attenuated [15]. *Salmonella *also contains non-fimbrial adhesins, encoded by chromosomal ***S**almonella *pathogenicity island 4 (SPI-4), of which the best characterized so far is SiiE, implicated in invasion at the apical side of polarized epithelial cells [16].

### Shigella flexneri

Very little is known about the adhesion structures that account for the initial contact between *Shigella *and a target cell. In contrast to other enteropathogenic bacteria that adhere to the small intestine, *Shigella *exhibits tropism for the colonic and rectal epithelia. This phenomenon could be explained by preferential adherence to human colonic mucin instead of small intestinal mucin observed in *Shigella dysenteriae 1 *[17]. Also, the fact that this binding was not inhibited by the presence of monosaccharides suggests that the receptor of *Shigella *adhesin is not a simple sugar.

**Figure 2:**
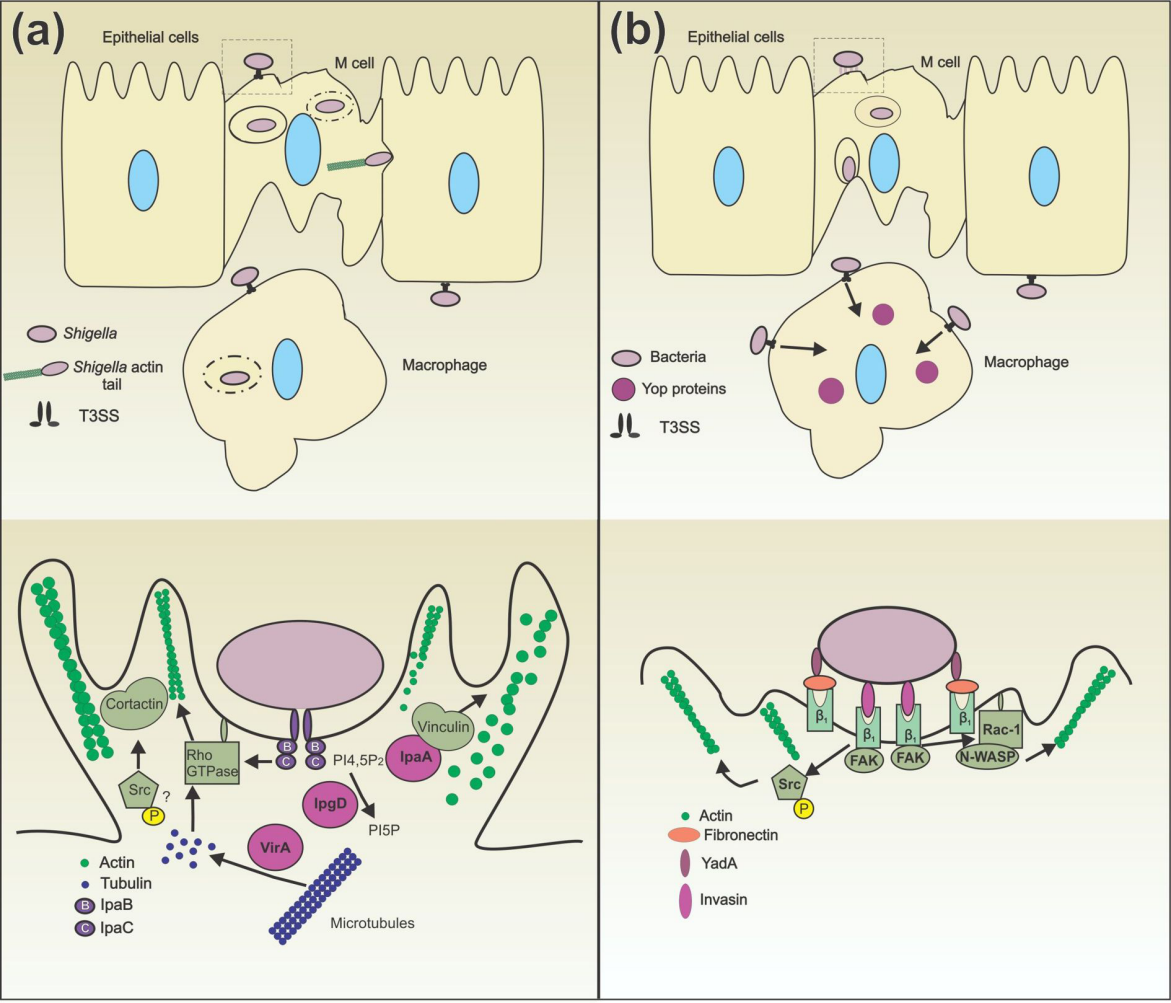
**Pathogenic mechanisms of (a) *Shigella flexneri *and (b) *Yersinia enterocolitica***. **(a) ***Shigella *interacts preferentially with M cells in the colonic and rectal epithelia (upper panel), activates its T3SS and secretes IpaB and IpaC to form a pore inserted into the host cell membrane (lower panel). The bacterium then delivers effector proteins through the translocon to the eukaryotic cell cytoplasm. IpaC also activates RhoGTPases that, together with activated Src, recruit cortactin, involved in actin filament reorganization. VirA promotes microtubule destabilization, leading to activation of RhoGTPases. IpgD generates PI(5)P, thus promoting cell survival through Akt. IpaA binds to vinculin and induces actin depolymerization. Once inside the host cell, *Shigella *leaves the vacuole for the cytoplasm and escapes to neighboring cells (upper panel). **(b) ***Yersinia *adheres initially to M cells (upper panel) by means of its adhesins: invasin, which binds β_1 _integrins directly; and YadA, which binds β_1 _integrins indirectly through fibronectin (lower panel). The interactions between the adhesins and integrins cause bacterial internalization following activation of FAK, Rac-1 and Src, which are involved in subtle actin cytoskeletal rearrangements. The vacuole containing the bacterium is transported towards the basolateral side of the M cell, where it is expelled into the dome region of the FAE (upper panel). When *Yersinia *interacts with phagocyte integrins, the T3SS is activated and a set of effector proteins (Yops) are translocated into the phagocyte cytoplasm. Yops are involved in the antiphagocytic and anti-inflammatory mechanisms used by *Yersinia *that lead to the formation of microabcesses in the FAE.

In *Shigella*, the bacterial proteins responsible for adhesion are the same as those that initiate the process of invasion. Following adherence, *Shigella *initiate activation of T3SS and secretion of effector proteins into the host cell (Fig. 2a). The genes encoding the translocon itself and the effector proteins, including four bacterial chaperons, are located in a 31-kb pathogenicity island within a large virulence plasmid (213 kb).

The protein IpaB binds to the receptor for hyaluronic acid, CD44, while IpaB complexed with IpaC binds to the fibronectin receptor, α_5_β_1 _integrin [18,19]. The complex IpaB/C then forms a structure resembling a pore inserted into the host cell membrane [20]; the pore links the T3SS translocon to the eukaryotic cell cytoplasm, and allows rapid delivery of other effector proteins (Fig. 2a). Apparently, the binding of IpaB to cholesterol present in lipid rafts is required for pore formation [21]. *Shigella *attracts membrane rafts to its site of entry, and these rafts tend to harbor signaling proteins, many of which will be targeted by the bacterium. The effector IpaD localizes to the tip of the T3SS needle; it has been suggested that this protein is involved in recognition and contact with the host membrane and controls the release of other effectors [22].

### Yersinia enterocolitica

After being ingested, *Yersinia *colonize the intestinal epithelium of the terminal portion of the ileum and proliferate in the underlying lymphoid tissue. Two non-fimbrial adhesins are crucial for adherence of *Yersinia *to host cells: invasin, encoded by the chromosomal gene *inv*, and YadA (***Y**ersinia ***ad**hesin **A**), encoded by the *Yersinia *virulence plasmid (pYV). Invasin is an outer membrane protein that binds a subset of β_1 _integrins (α_3_β_1_, α_4_β_1_, α_5_β_1_, α_6_β_1 _and α_v_β_1_) through its extracellular, C-terminal, portion. Although invasin lacks an RGD domain and shares no sequence homology with fibronectin, the natural ligand of β_1 _integrins, it binds α_5_β_1 _in the same residues and with a 100-fold higher affinity than fibronectin [23]. While β_1 _integrins are restricted to the basolateral surface of enterocytes [24], M cells express them on their apical surface, where they are available for bacterial adherence. The interaction of invasin with an integrin causes the agglomeration of integrin receptors over the bacteria-host interface, thus accounting for the "zipper" mechanism of *Yersinia *internalization observed by scanning electron microscopy. In contrast to invasin, YadA binds diverse extracellular matrix components, such as collagen, laminin and fibronectin, thus indirectly mediating integrin binding [25] (Fig. 2b).

## Cellular Invasion

Following adhesion, infection by *Salmonella*, *Shigella *and *Yersinia *involves internalization, a process induced by the bacteria themselves. EPEC, in contrast, remain extracellular, although some reports point to an ability to invade due to the action of the effector protein EspG, which presumably interacts with tubulin [26], like the VirG protein of *Shigella *(see below).

In this section, we will consider the major mechanisms by which *Salmonella*, *Shigella *and *Yersinia *trigger their own uptake.

### Salmonella enterica

Following adhesion between *Salmonella *and host epithelial cell or M cell, the T3SS encoded by chromosomal ***S**almonella *pathogenicity island 1 (SPI-1) is activated. The bacterium then injects at least 12 effectors that trigger bacterial uptake.

***S**amonella ***i**nner **p**roteins (Sip) A and C are probably the first proteins delivered through the T3SS to the host cell [27](Fig.1b). Within the host cell, SipC localizes to the plasma membrane, where it aids in the translocation of other *Salmonella *effectors and initiates actin nucleation. The C-terminal domain nucleates actin and the N-terminal domain of SipC bundles it, anchoring the resulting actin filaments to the cell surface below the bacterium [28]. The injected SipA acts in synergy with SipC, as SipA binds to and stabilizes the F-actin filaments, and physically blocks the action of an actin depolymerising factor (ADF/cofilin).

The ***S**almonella ***o**uter **p**roteins (Sop) E (SopE1 and SopE2), SopB and SptP are also delivered (in a SipC-dependent way) through the T3SS translocon into the host cell, where they are targeted to the plasma membrane [27]. SopE and SopE2 act as guanine exchange factors (GEFs) for the Rho monomeric GTPases RhoG, Rac1 and Cdc42 [29], which are involved in the formation of filopodia extensions and lamellipodia structures. SopB (also known as SigD) also activates Cdc42, by a poorly understood mechanism, and RhoG by activating the endogenous GEF [30]. SopB is an inositol polyphosphate phosphatase that interferes with phosphoinositide phosphate and inositol phosphate metabolism [31]: SopB eliminates phosphatidylinositol-3,5-biphosphate (PtdIns(3,5)P_2_), PtdIns(4,5)P_2 _and PtdIns(3,4,5)P_3_, generating PtdIns(3)P at the site of bacterial invasion; PtdIns(3)P is probably the activator of the GEF for RhoG, and is retained in the *Salmonella*-containing vacuole [32].

Activation of RhoG, Rac1 and Cdc42 by *Salmonella *leads to activation of WASp, which, complexed with monomers of globular actin and Arp2/3, initiates nucleation of actin and its polymerization into actin filaments. The induced cytoskeletal rearrangements cause membrane ruffling and culminate in macropinocytosis of bacteria by the host epithelial cell [33]. The fact that *Salmonella *mutants defective in SopE, SopE2 or SopB remain invasive and only mutants lacking all three proteins display a non-invasive phenotype illustrates the redundancy of the *Salmonella *infection mechanisms [31].

Takeuchi (1967) had already observed that the epithelial cell soon recovers its normal shape following macropinocytosis of bacteria. This recovery is brought about by the effector protein SptP, which acts as a GTPase-activating factor for the RhoGTPases targeted by the SopE and SopB proteins, thereby switching off the signal [34].

### Shigella flexneri

After having formed a pore in the membrane of M or epithelial cells, the IpaB/C complex triggers the initial events in actin polymerization. The C-terminal domain of IpaC activates Cdc42, which in turn activates Rac1, thereby inducing the formation of filopodial and lamellipodial extensions [35]. *Shigella *also somehow activates the tyrosine kinase Src (pp60c), which is implicated in recruitment by phosphorylation of actin-associated proteins such as cortactin [36].

The receptor for hyaluronic acid, CD44, once activated by IpaB, recruits ezrin to the site of entry. Ezrin recruitment seems to depend on Rho, whose activation is enhanced by the IpaB-CD44 complex [37]. Ezrin associates with F-actin and functions as a membrane-cytoskeleton linker in the filopodial structures. Together, tyrosine kinases and small GTPases reorganize the actin cytoskeleton into entry structures similar to the focal adhesions in the host cell.

VirA is a protein encoded by a virulence plasmid and secreted in an Ipa-independent manner; it interacts with and destabilizes α/β-tubulin heterodimers, probably stimulating Rac-1 activity, which thus promotes the formation of lamellipodial structures around bacterial entry foci [38].

Two other T3SS-secreted proteins are IpgD and IpaA. IpgD is a phosphatase with homology to SopB/SigD of *Salmonella*; it generates PtdIns(5)P from PtdIns(4,5)P_2 _at the site of entry, and PtdIns(5)P in turn activates the PI-3 kinase/Akt pathway, thus contributing to cell survival [39]. IpaA complexes with the focal adhesion protein vinculin, and induces vinculin capping activity at the fast growing ends of F actin, thus promoting F actin depolymerization [40].

These events are summarized in Fig. 2a.

### Yersinia enterocolitica

Activation of integrin receptors by invasin and/or YadA triggers several intracellular signals, which are very similar to the events that precede cell division: the cytoplasmic tail of β1 chain interacts with focal adhesion kinases (FAKs), Src and the Rac-1-Arp2/3 complex, which in turn trigger slight cytoskeleton rearrangements needed for bacterial uptake [41] (Fig. 2b, lower panel). This clustering or zipper mechanism of invasion contrasts with the trigger mechanism used by *Salmonella *and *Shigella*.

*Yersinia *species also hijack host cell phosphoinositide metabolism for their uptake. Rac-1 recruits, and Arf6 activates, the type I phosphatidylinositol-4-phosphate-5-kinase (PtdIns(4)P(5)Kα), which forms PtdIns(4,5)P_2 _at the entry site, where PtdIns(4,5)P_2 _may regulate phagocytic cup formation by coordinating membrane traffic and controlling F-actin production [42].

*Yersinia *internalized by M cells remains intracellular, bounded by a polymerized actin-coated vacuole. The bacterium survives inside the vacuole, even though it does not replicate. This vacuole is then transported from the apical to the basolateral side of the M cell, where the bacterium is expelled and exposed to the dome region of the FAE, which is densely populated by dendritic cells, macrophages and lymphocytes (Fig. 2b, upper panel). In this way, *Yersinia *crosses the epithelial barrier. However, *Yersinia *species have evolved a dual strategy for avoiding destruction and establishing infection: an antiphagocytic and an anti-inflammatory strategy.

When *Yersinia *invasin interacts with phagocyte integrins, the extracellular adherent bacteria transfer a set of pathogenic factors, known as Yops (***Y**ersinia ***o**uter **p**roteins) through a pYv-encoded T3SS to the target cell, and this inhibits the uptake of *Yersinia *by interrupting the phagocytic pathway [43]. The T3SS apparatus is necessary for injection of the effectors (YopE, YopP, YopT, YopH, YopO and YopM) into the host cell [44]. Three translocator proteins are known to be required for the injection: YopB, YopD, which are believed to form a pore in the host cell membrane, and LcrV (also called V-antigen), which is localized to the tip of the needle and acts as a scaffold protein for the correct insertion of the pore formers in the membrane [45]. LcrV is also an important anti-inflammatory agent, which has been implicated in suppression of NF-κB and interferon-γ and the secretion of inhibitory interleukin IL-10 by macrophages [46].

YopH, YopE, YopT and YopO act indirectly on the actin cytoskeleton. YopH is a tyrosine phosphatase that dephosphorylates several macrophage proteins involved in focal adhesion, so opposing the phagocytic pathway induced by the invasin-integrin interaction [47]. Moreover, YopH rapidly blocks the neutrophil calcium signaling pathway induced by invasin-integrin binding, thereby inhibiting their degranulation [46]. YopE has GAP activity and there is evidence that it selectively inactivates Rac but not Rho or Cdc42 [48]. This inhibition of Rac activity ultimately leads to the arrest of membrane ruffling, otherwise an important step in engulfment. Aili and colleagues have shown that *Yersinia *devoid of YopE has high levels of Yop translocation into HeLa cells. This suggests that the GAP activity of YopE is also important in the modulation of pore formation, since YopE plays a role as a feedback inhibitor of Yop translocation [49]. YopT also has an anti-phagocytic role, since it removes the geranylgeranylated C-terminal cysteine of RhoA; once this occurs the enzyme is released from the plasma membrane and inactivated, so preventing actin reorganization [50]. The effector protein YopO, also known as YpkA (*Yersinia *protein kinase A), possesses a C-terminal actin-binding domain necessary for YopO activation, and a domain that mimics Rho and Rac guanine nucleotide dissociation inhibitors (GDI) [51] and maintains these factors in their GDP-bound inactive states, while its N-terminal region harbors a domain with serine/threonine kinase activity that phosphorylates a critical serine residue on G_αq _protein, blocking downstream calcium signaling [52]. Apparently, both the GDI and kinase activities of YopO are involved in disruption of the host cytoskeleton.

YopM, in contrast, has no enzymatic domain; instead it acts like a scaffolding protein, regulating the activity of two host cell kinases, PRK2 and RSK1, both involved in pathways signaling cell survival and proliferation. Association of YopM with PRK2 increases its kinase activity, which in turn activates RSK1. Once activated, the YopM-kinase complex may phosphorylate a still unknown substrate [53].

YopP, a homologue of YopJ, orchestrates an anti-inflammatory process by exerting an inhibitory effect on both the mitogen-activated protein kinase (MAPK) and NF-κB signaling pathways [54]. YopJ has an acetyl transferase activity that modifies the activation loop of the MAPK and Iκβ kinases^2 ^(see Appendix), thereby preventing their phosphorylation and subsequent activation [55]. Inhibition of the MAPK and NF-κB signaling pathways results in rapid apoptosis of the macrophages, which is important for establishing a systemic infection.

In addition to their action on cell adhesion, the surface factors YadA and Ail may be involved in serum resistance, probably by capturing the C4b-binding protein that down-regulates the classical and lectin complement pathways [56]. Later in infection, when *Yersinia *interacts with epithelial cells, *Yersinia *invasin is able to stimulate NF-κB synthesis and trigger the production of pro-inflammatory cytokines, such as IL-8, via MAPK. The YadA-ECM-β_1 _interaction also leads to IL-8 production, although this occurs via a different MAPK [57].

IL-8 and other cytokines activated by *Yersinia *are chemoattractants and promoters of PMNs and macrophages. These cells are recruited to infection sites, leading to disruption of the epithelial barrier, and exposing the integrins localized on the basolateral sides of enterocytes, causing bacterial dissemination [57]. Ulceration and necrosis of the tissue subjacent to the FAE are also characteristic of *Yersinia *infections. Systemic dissemination begins 24-48 h after infection and may be enhanced by bacterial phagocytosis; the bacteria travel through the bloodstream towards the spleen and liver, where they replicate in microabscesses, forming microcolonies resistant to phagocytosis.

## Intracellular Lifestyle

Here we will discuss how intracellular *Salmonella *and *Shigella *avoid being digested; while *Salmonella *remains in the vacuole and adapts to the harsh environment, *Shigella *escapes from the phagosome into the cytoplasm and neighboring cells.

### Salmonella enterica

Once intracellular, either in M cells, epithelial cells or phagocytes, *Salmonella *is enclosed in an acidic compartment called a *Salmonella-*containing vacuole (SCV), in which the bacterium alters the endocytic pathway in order to avoid being destroyed, and replicates.

As mentioned above, *Salmonella *invasion is accompanied by the formation of PtdIns(3)P and disappearance of PtdIns(4,5)P_2 _at the site of entry due to the action of SopB [32]. PtdIns(4,5)P_2 _depletion improves actin depolymerization and facilitates membrane fluidity. The local region of membrane invaginates to form PtdIns(3)P-enriched vacuoles; PtdIns(3)P recruits early endosomal markers, including the GTPase Rab5 and EEA1 (early endosomal antigen 1), which promote fusion between the SCV and empty vesicles formed during bacterial invasion, creating a spacious habitat for *Salmonella *[32]. These early markers are quickly replaced by late endosomal markers (Rab7, LAMP-1, LAMP-2 and LAMP-3) and the vacuolar H^+^-ATPase (V-ATPase), which decreases the pH inside the SCV. Phagosome acidification by the V-ATPase is related to the maturation of lysosomes and the bactericidal function of lysosomal hydrolases. SCV acidification is a key stimulus for *Salmonella *to express and assemble, intracellularly, the second pathogenicity island-encoded T3SS (SPI-2 T3SS), approximately three hours after infection [58]. One study has revealed that SCV acidification in epithelial cells containing *Salmonella *occurs more slowly than in vacuoles containing heat-killed *Salmonella *or non-pathogenic *Escherichia coli *(expressing invasin). Apparently, this delay is independent of both *Salmonella *T3SSs, yet the delayed acidification seems to allow the bacteria to adapt to the new intracellular environment by decreasing the activity of lysosomal hydrolases [59].

According to a recent report by Yu and colleagues [60], the SPI-2 T3SS is assembled at the low pH of the SCV, but secretion of a second set of effectors through the SCV membrane depends on sensing the neutral pH of the host cytosol. Effectors SseF and SseG colocalize with the microtubule cytoskeleton and interfere with its organization by inducing bundle formation. SseF and SseG, together with SifA (see below), affect the microtubule network and influence SCV positioning, helping to guide the *Salmonella *towards the perinuclear region, next to the Golgi complex [61,62]. This positioning facilitates interaction of the SCV with the endocytic and secretory pathways, allowing the pathogen to acquire extra membrane for expansion of the SCV, and nutrients to promote intracellular survival.

Four to six hours after entry, the SCV is characterized by the presence of long membrane structures, called ***S**almonella-***i**nduced **f**ilaments (Sifs) that extend from the SCV. Sifs are enriched in lysosomal membrane glycoproteins such as LAMP-1, and their formation is dependent on host cell microtubules [63]. These filaments increase the size of SCVs by incorporating into them PtdIns(3)P-enriched endosomes formed during *Salmonella *entry but devoid of bacteria, and thus provide space for *Salmonella *to replicate. SseF and SseG also somehow contribute to Sif formation, although to a lesser extend than SifA [64].

During its maturation, the SCV interacts with host endocytic-recycling regulators to regulate vacuole membrane renewal [65]. The lipid composition of the SCV may be modulated by the SPI-2 T3SS effector SseJ, which has a high degree of similarity to acyltransferases/lipases and its enzymatic function is likely to oppose that of SifA, down-regulating Sif formation [66]. In addition, the effectors SspH2 and SseI were observed to colocalize with the F-actin cross-linkers filamin and profilin, and are thus apparently involved with the dynamics of the actin cytoskeleton around the SCV [67]. Actin filaments in the vicinity of the SCV membrane are assembled under the influence of the bacteria from within the vacuole in an SPI-2 T3SS-dependent manner, and seem to maintain the integrity of the vacuole and/or recruit extra membrane that is necessary to increase the membrane surface area to enclose the replicating bacteria [68].

Inside the SCVs within phagocytes, the pathogen may inhibit, in an SPI-2-dependent manner, production of reactive oxygen species by preventing assembly of the NADPH oxidase complex [69], and of reactive nitrogen species by removing inducible nitric oxide synthase from the vicinity of the SCV [70]. It is possible that the actin meshwork and microtubule organization around the SCV affect the trafficking of these enzymes by physically blocking their colocalization with the vacuole.

Both SPI-1 and SPI-2 T3SSs expressed by *Salmonella *are able to induce macrophage death, although by distinct mechanisms and at different times of infection. The SPI-1 T3SS effector protein SipB activates caspase-1, triggering programmed pro-inflammatory cell death (pyroptosis) within 45 minutes of infection. Alternatively, macrophage death mediated by SipB may be simply a consequence of that effector's ability to induce cell lysis. Delayed macrophage death (up to 24 h) may occur in an SPI-2 T3SS-dependent manner, although by a still unknown mechanism [71]. Once activated, caspase-1 cleaves the inactive precursors of the cytokines interleukin 1β (IL-1β) and interleukin 18 (IL-18) to their mature, active forms. IL-1β and IL-18 are necessary to trigger the intestinal inflammation response characteristic of salmonellosis. Another pro-inflammatory cytokine generated during the infection is IL-8, activated by Cdc42 after it is activated by the SopE, SopE2 and/or SopB effectors [30].

The inflammatory response generated against *Salmonella *may compromise the intestinal barrier, allowing bacteria to cross it and encounter the phagocytes that have been attracted to the infection site. Once inside phagocytes, the bacteria survive and replicate, and some *Salmonella *species spread from Peyer's patches to the mesenteric lymph nodes, creating a systemic infection. Apoptosis of epithelial cells may also contribute to the observed disintegration of the intestinal epithelia in salmonellosis, since HT-29 human intestinal cells infected with *S. dublin *undergo delayed (not earlier than 24 h p.i.) apoptosis preceded by caspase-3 activation, in a process dependent on effectors encoded by the SPI-2 loci [72].

Although efficient colonization of the host is advantageous for the pathogen, *Salmonella *appears to express antivirulence modulators during systemic infection: using a murine model, Gal-Mor *et al*. (2008) [73] have shown that when *Salmonella *Typhimurium reaches the intestinal tract, it expresses ZirT, an outer membrane protein with a β-barrel conformation, which translocates the protein ZirS; both proteins have been implicated in reducing the bacterial load in the infected mice.

### Shigella flexneri

Once internalized by M cells of the FAE, *Shigella*, unlike *Salmonella*, disrupts the vacuole membrane in a process dependent on the IpaB and IpaC invasins and escapes into the host cell cytoplasm, where it proliferates. Once present in the cytoplasm, the pathogen accumulates polymerized actin at one pole of the bacterium, forming a "tail" that propels it through the cytoplasm and enables it to invade neighboring epithelial cells. The polymerization of actin depends on the action of IcsA (intracellular spread A)/VirG, an outer membrane protein that has a polar distribution on the bacterial surface.

IcsA/VirG recruits host cell cytoskeletal proteins; it interacts with N-WASP and increases its affinity for Arp2/3, forming the complex IcsA/N-WASP/Arp2/3 that induces actin nucleation. IcsA/VirG also recruits vinculin, and although vinculin is not necessary for movement of the pathogen, it may contribute to the polymerization of actin. Moreover, actin depolymerization factor (ADF)/cofilin, capping proteins and profilin are recruited to stabilize the tail. *Shigella *intracellular movement also depends on the tubulin-degrading activity of VirA, which opens the way through the microtubule network for *Shigella *[38]. When the bacterium eventually reaches the inner face of the basolateral membrane of the host cell, a finger-like protrusion is formed which is endocytosed by a neighbouring epithelial cell. Once endocytosed, the bacterium again escapes from the vacuole, proliferates in the host cell cytoplasm and invades new cells, thus advancing the course of disease. This process damages the tight junctions between adjacent cells, damage that is worsened by altered expression of tight junction-associated proteins induced by the pathogen [12]. Disruption of the integrity of the colonic mucosa leads to massive invasion of bacterial cells by exposing the basolateral sides of colonocytes.

As a consequence of the tissue invasion, epithelial cells start to produce a set of pro-inflammatory cytokines such as IL-8, which together with the infecting *Shigella*, strongly attracts polymorphonuclear (PMN) cells from the sub-epithelial layer to the intestinal lumen. The PMN infiltrate may exacerbate bacterial invasion at an early stage, but it ultimately leads to resolution of the infection, since the PMN cells do not allow the pathogen to cross the FAE and spread into the bloodstream. The negative consequences of PMN infiltration are severe inflammation and massive destruction of the colonic mucosa, causing mucosal abscesses and ulcers, the clinical characteristics of shigellosis [74].

After traversing M cells, *Shigella *exposes itself to an area of the FAE intensively populated with macrophages and dendritic cells. There, like *Salmonella*, it triggers macrophage pyroptosis: the translocated IpaB activates caspase-1, which in turn cleaves IL-1β and IL-18 to their mature, active forms [75]. Besides requiring membrane cholesterol [76], activation of caspase-1 by IpaB appears to require Ipaf, a Nod-like receptor localized on plasma and endosomal membranes and implicated in bacterial recognition [77]. IL-1β release causes rupture of the epithelial barrier, enhancing bacterial dissemination, which in turn increases inflammation and subsequent tissue destruction. Conversely, the parallel release of IL-18, a potent interferon-γ inducer, aids host killing of *Shigella *by activated immune cells, thus demonstrating a protective role in shigellosis [78]. *Shigella *also secrete effectors through the T3SS that are engaged in down-regulating inflammation and these effectors may facilitate the early steps of infection. IpaH and OspF accumulate in the nucleus: IpaH binds to U2AF, a mammalian splicing factor, affecting the expression of pro-inflammatory cytokines [79], and OspF dephosphorilates and inactivates MAPKs [80]. A third effector, OspG, seems to prevent IκBα degradation, so interfering with the NF-κB activation pathway.

## Conclusions

Knowledge of the pathogenesis of diarrheal diseases due to bacteria has grown greatly over the past two decades. The study of the bacterial pathogens described in this review has greatly illustrated the sophisticated mechanisms pathogens have developed to efficiently mimic and manipulate several host signaling pathways. In consequence, they succeed in colonizing the gut epithelium: EPEC do so by adhering tightly to epithelial cells and remaining extracellular; *Salmonella, Shigella *and *Yersinia*, in contrast, invade the gut epithelium. While *Salmonella *and *Shigella *are phagocytosed by macrophages and provoke an inflammatory response that disrupts the epithelial barrier, *Yersinia *avoids macrophage phagocytosis and inhibits the host inflammatory response. In all cases, the bacteria multiply and are able to leave the host and be transmitted to another host. Understanding at the molecular level of the systems developed by pathogenic bacteria may allow us to target essential bacterial effectors in novel therapeutic approaches to the infections. On the other hand, the lack of epidemiological studies identifying the predominant strain in a given endemic region, and the fact that diarrheal diseases are not always caused by a single etiological agent, certainly make such approaches more difficult to put into practice.

Regardless of its direct clinical application, the study of diarrheagenic bacteria as models of longstanding host-pathogen interactions has revealed how eukaryotic cells react under stress and how they mount an inflammatory response; such knowledge contributes to our understanding of infectious diseases, and also of non-infectious diseases. Moreover, the study of the diarrheagenic bacteria reviewed here has been giving new approaches in the investigation of cellular microbiology of other bacterial pathogens.

## Competing interests

The authors declare that they have no competing interests.

## Authors' contributions

RSR draft the manuscript; RSR and FH wrote and revised the manuscript; both authors read and approved the final manuscript.

## Appendix

1. Actin filaments (F-actin) are polymers of globular actin (G-actin) that associate with other proteins to form a cellular cortex beneath the plasma membrane. This net provides mechanical support for the cell membrane and allows changes that generate lamellipodia, filopodia and focal adhesion structures controlled by different members of the Rho GTPase family.

2. The family of NF-kB transcription factors is controlled by the binding of an inhibitor protein, named IkB, which sequesters monomers of NF-kB in the cytoplasm. NF-kB activation requires removal of the IkB from the complex through the action of an IkB kinase that phosphorylates the regulatory region of the inhibitor. The phosphorylated IkB conjugates with ubiquitin and is subsequently proteolytically degraded. Dimeric NF-kB is then able to move to the nucleus and bind to target DNA, thereby controlling the expression of a wide range of genes involved in the inflammatory response.
